# Incorporating GO/KEGG Functional Annotations Improves the Accuracy and Stability of Genomic Prediction Across Diverse Beef Cattle Populations

**DOI:** 10.3390/ani16121776

**Published:** 2026-06-08

**Authors:** Le Zhou, Lin Zhu, Fengying Ma, Mingjuan Gu, Risu Na, Wenguang Zhang

**Affiliations:** 1 College of Animal Science, Inner Mongolia Agricultural University, Hohhot 010010, China; zxcvbnm8880314@163.com (L.Z.); zhulinynacxhs@163.com (L.Z.); fengyingma1997@163.com (F.M.); gmj0119@yeah.net (M.G.); narsanjargal@imau.edu.cn (R.N.); 2 Inner Mongolia Engineering Research Center of Genomic Big Data for Agriculture, Inner Mongolia Agricultural University, Hohhot 010010, China; 3 College of Life Science, Inner Mongolia Agricultural University, Hohhot 010010, China

**Keywords:** functional annotations, cross-population prediction, admixture proportion, G Matrix, GO, KEGG

## Abstract

GS in beef cattle faces challenges in cross-population prediction. Blind expansion of reference populations can reduce accuracy because of genetic noise, and traditional models such as GBLUP ignore functional heterogeneity of SNPs. In this study, we used three simulated beef cattle populations with different genetic relationships and a single trait corresponding to birth weight (heritability 0.42) to evaluate the effect of functional annotations on cross-population GS. We compared GBLUP, ssGBLUP and wGBLUP using either all SNPs or SNP sets annotated by GO and KEGG. Mixed reference populations were constructed with multidimensional scaling (MDS)- and fixation index (FST)-based screening. Functionally annotated SNPs increased cross-population prediction accuracy and stability compared with generic SNPs. In the population with close genetic relatedness (PopB), GO-wGBLUP achieved a prediction accuracy of 0.55–0.60 at a 10% reference proportion, higher than GBLUP (about 0.50) and ssGBLUP (about 0.52). In the population with high genetic differentiation (PopA), KEGG-wGBLUP showed a smaller loss of accuracy when the reference proportion increased from 10% to 20% (6.7% decline, from 0.45 to 0.42) than GO-wGBLUP (10% decline, from 0.50 to 0.45) and GBLUP (20% decline, from 0.35 to 0.28). Across scenarios, functional SNP sets reduced the loss of accuracy due to reference population expansion from 20.0% in GBLUP to 12.5% in GO-wGBLUP. These results indicate that wGBLUP combined with GO or KEGG annotations can improve the accuracy and robustness of cross-population GS for beef cattle birth weight traits. GO-based models are more suitable for closely related populations, whereas KEGG-based models are more suitable for highly differentiated populations. The proposed framework provides a practical reference for multi-population GS design in beef cattle breeding.

## Simple Summary

Genomic selection (GS) in beef cattle faces accuracy declines in cross-population prediction due to genetic noise and unaccounted SNP functional heterogeneity. We integrated GO/KEGG functional annotations into genomic selection models to prioritize trait-relevant variants. Results showed annotated SNPs improved prediction accuracy and stability, with weighted GBLUP (wGBLUP) performing optimally. Gene Ontology (GO) annotations benefited closely related populations, while Kyoto Encyclopedia of Genes and Genomes (KEGG) suited divergent groups, mitigating performance loss from reference population expansion. This strategy provides a robust framework for multi-population genomic selection in beef cattle breeding.

## 1. Introduction

GS has become a core approach driving the technological innovation of modern livestock and poultry breeding, owing to its advantage of enabling accurate early-stage selection independent of phenotypic data [1]. It is particularly promising for improving complex traits with low to moderate heritability, including fertility and meat quality. Specifically, beef cattle meat quality traits generally have low to moderate heritability, while some traits, especially marbling, can be moderately to highly heritable depending on the breed and population. In addition, it offers an effective approach to shortening breeding cycles and reducing the high costs of phenotypic measurements in conventional breeding programs [2,3]. These constraints limit the size of within-breed reference populations and motivate the use of multi-breed or across-breed genomic selection strategies.

With the growing industrial demand for diversified breed improvement, the limitations of single-breed genomic prediction models have become prominent. Multi-breed or cross-breed genomic selection builds a joint reference population by combining genotypic and phenotypic information from multiple breeds. By exploiting genetic relationships and shared variants across breeds, it increases genetic diversity in the reference set and improves the power to detect rare variants and common quantitative trait loci (QTL) [4]. This strategy offers a new approach for local or minor breeds with small population sizes and difficulties in establishing reference populations. Multiple studies have verified its effectiveness: constructing reference populations among genetically closely related breeds yields significantly higher prediction accuracy than other combinations [4]; for individuals without close genetic relationships, multi-breed evaluation can also improve the prediction precision of their estimated breeding values (EBV) [5]; for local breeds, multi-breed evaluation not only boosts prediction accuracy but also helps reduce evaluation bias [6]. The fundamental reason lies in the shared linkage disequilibrium (LD) structures and similar QTL effects among populations.

Among the current genomic prediction methods, genomic best linear unbiased prediction (GBLUP) is widely used in cattle breeding programs [7,8,9]. GBLUP uses a genomic relationship matrix constructed from dense SNP markers to predict genomic breeding values under the infinitesimal model assumption. Because of its simple implementation and robustness, GBLUP is often regarded as the baseline model for evaluating genomic selection strategies.

However, the implementation of cross-population genomic selection in beef cattle still faces key constraints, particularly limited prediction accuracy and inadequate robustness [6]. Beef cattle populations feature complex genetic backgrounds and significant structural differentiation, leading to strong genetic relationship heterogeneity between reference and target populations. Blind merging of population data with large genetic differences may introduce genetic noise and dilute effective signals. In addition, GBLUP assumes equal effects for all SNP markers and does not explicitly account for the heterogeneous functional roles of genetic variants, which may limit its ability to capture conserved functional variation across populations [10]. Prediction performance strongly depends on the genetic architecture of the trait, including the distribution of QTL and the consistency of their effect sizes, as well as on the genetic distance between breeds [11]. Even when using models with population structure correction, interference cannot be completely eliminated if breed-specific allelic effects and LD pattern heterogeneity are not fully considered [12].

The key to breaking these bottlenecks lies in acquiring more comprehensive genetic variation information and more accurate biological prior knowledge. Conventional medium-density SNP chips often fail to maintain a stable LD relationship with QTLs in cross-breed analyses [13]. Integrating whole-genome sequencing (WGS) data with functional annotation information offers a new approach. WGS data can fully cover rare and potential causal variants and strengthen the association between markers and QTLs, yet it is characterized by high data noise and computational cost, necessitating preselection strategies (e.g., focusing on coding regions or screening based on GWAS results) to reduce redundancy [14]. The core value of functional annotation is to provide biological priors for distinguishing functional variants from neutral ones, thereby supporting the accurate identification of causal variants [15].

Integrating functional annotation into genomic prediction models and assigning differential weights to markers based on their functional categories can concentrate statistical power on biologically relevant genomic regions, reduce interference from neutral variants, and improve prediction efficiency [16]. Incorporating functional annotation into variable selection models (e.g., BayesCπ) can significantly enhance the ability to detect functional variants, and in multi-population analyses, it can also help narrow the confidence intervals of QTLs [10]. Preliminary applications have demonstrated its promise. In multi-breed cattle evaluations, incorporating functional annotations into genomic prediction models markedly improved cross-population prediction accuracy for specific carcass and milk production traits [17]. In pigs, models that integrate prior information on QTL regions also increased prediction accuracy and showed stable performance in admixed populations [18]. However, the standardized integration of functional annotation into genomic prediction models is still difficult. To address this gap, we followed a simple and reproducible framework. First, we selected annotation resources that match the species and the biology of the target trait. Second, we defined genomic regions around annotated genes as candidate functional regions. Third, we constructed functional genomic relationship matrices from SNPs within these regions and compared them with conventional genomic matrices. This framework can be transferred to other populations and traits by adapting the annotation resources and gene region definitions. The selection of annotation information needs to be dynamically tailored to the population genetic background and the genetic architecture of the trait. At present, a standardized and unified workflow for this process has not been established [18].

Based on GBLUP, ssGBLUP, and wGBLUP models, we reconstruct the genomic relationship matrix by integrating GO and KEGG annotations. We first partition SNPs into functionally defined sets according to their GO terms and KEGG pathways, and then construct multiple genomic relationship matrices from these sets. In this way, variants located in functionally important genes or pathways make a greater contribution to the genomic relationships. We then systematically evaluate the prediction performance of each model under different population genetic relationships, degrees of genetic differentiation, and reference population sizes. On the basis of traditional GBLUP, single-step GBLUP and weighted GBLUP models, we will reconstruct the genomic relationship matrix by integrating GO/KEGG annotations and systematically analyze the prediction performance of each model under different population genetic relationships, genetic differentiation degrees and reference population sizes. This study intends to clarify the application rules and efficiency-increasing mechanisms of the two functional annotation systems in cross-population prediction of beef cattle, and elucidate the improvement effect of functional annotations on prediction performance. Thus, it provides a scientific basis for optimizing the construction strategy of multi-breed reference populations and model selection, and promotes the precise application of multi-breed joint genomic selection technology in beef cattle.

## 2. Materials and Methods

### 2.1. Data Simulation

We used QMSim Version 1.10 [19] to simulate beef cattle genomic datasets. By modifying key parameters related to linkage disequilibrium (LD) formation in QMSim, including the effective population size of ancestral populations, mutation rate, and recombination rate, we generated three beef cattle populations (PopA, PopB, and PopC) with distinct LD patterns. Each population was simulated in five independent replicates, and each replicate produced a complete dataset of breeding animals. Phenotypes were sampled from a normal distribution with mean 0 and variance 1, and the overall mean was determined by the population fixed effect.

#### 2.1.1. Population Structure

In this study, PopA, PopB and PopC represent three hypothetical beef cattle populations rather than specific named breeds. They were designed to mimic populations with different demographic histories and distinct linkage disequilibrium patterns, and thus different levels of genetic differentiation relative to PopA. PopA corresponds to a population with long-term small effective size followed by expansion, PopB mimics a population that experienced a bottleneck and then expansion, and PopC represents a population with an early expansion followed by a contraction. These scenarios are intended to reflect cross-population genomic selection situations between populations with close, moderate and distant genetic relationships.

Three beef cattle populations (PopA, PopB, and PopC) with distinct LD patterns were simulated. Each population followed four consecutive stages: historical evolution, establishment of initial LD and mutation–drift equilibrium, population expansion, and final population simulation. The selection strategy in the final stage was the same for all populations and was based on phenotypic performance and best linear unbiased prediction (BLUP) of EBV. The missing-record rate for both bulls and cows was fixed at 0.05, whereas other parameters and demographic trajectories differed among the three populations.

PopA represented a continuous expansion pattern from a stable ancestral population. Its historical population consisted of 200 individuals maintained for 1000 generations, followed by an expansion to 1000 individuals (100 males and 900 females) over the next 95 generations to establish the base equilibrium. After further expansion, the final simulation was performed for 10 generations with 200 selected bulls and 2800 selected cows, using replacement rates of 60% for bulls and 30% for cows.

PopB followed a shrinkage-then-expansion pattern from a stable initial population. The historical population size was reduced from 500 to a stable size of 200 individuals over 1000 generations, and then expanded to 1000 individuals (100 males and 900 females) during the subsequent 95 generations to reach the base equilibrium before further expansion. The final 10-generation simulation was carried out with 220 selected bulls and 2335 selected cows, with replacement rates of 50% for bulls and 30% for cows.

PopC presented a continuous population shrinkage pattern. The initial stable population with 1000 individuals remained unchanged for 1000 generations, and then gradually decreased to 200 individuals, including 100 males and 100 females, within the subsequent 95 generations to form the basic balanced population. The final 10 generations were simulated using 200 selected sires and 2800 selected dams, with the replacement rate set at 50% for sires and 20% for dams.

**Figure 1:**
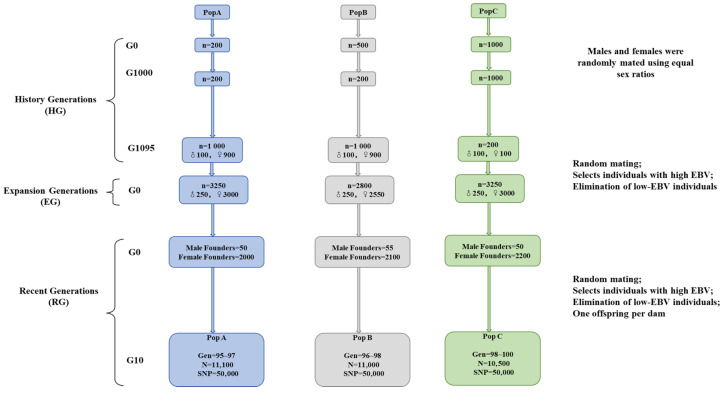
Schematic representation of the simulated population structure. Note: Three beef cattle populations with significantly different LD patterns were simulated in this study, with distinct population evolutionary patterns: PopA exhibited a continuous expansion pattern, PopB showed a fluctuating evolutionary pattern of expansion-shrinkage-expansion, and PopC displayed a continuous shrinkage pattern. The figure presents core parameters including the total number of individuals (n) per generation and the number of male (♂) and female (♀) individuals per generation for each population. In the figure, “Gen” denotes the generation range of the populations used for analysis, “N” represents the total number of individuals included in subsequent analyses, and “SNP” indicates the total number of SNP markers incorporated into the analysis.

During the expansion phase of all populations, random mating was applied, and each dam produced one offspring per generation for 10 consecutive generations. The simulation parameters for the three populations are summarized in Table 1, and the historical, base, and current population structures are illustrated in Figure 1.

#### 2.1.2. Genome

Based on the bovine genome assembly version ARS-UCD1.2 released on Ensembl (Ensembl genome browser 115), we simulated 29 pairs of autosomes for beef cattle with a total genetic length of 2715.85 cM. This design aimed to approximate realistic genomic structure by accounting for the physical distances between markers and QTL. We generated 50,000 biallelic SNP markers that were uniformly and randomly distributed across the genome, with the number of markers on each chromosome proportional to its length. All markers were assumed to have no direct effect on the target trait.

The simulated genome contained 725 QTL. The number of recombination events followed a Poisson distribution with a mean of 1 per Morgan, and crossover positions were randomly distributed along the chromosomes. QTL effects for the production trait were introduced in the last generation of the historical population. The trait heritability was set to 0.42, consistent with reported estimates for birth weight in beef cattle [20]. QTL effects were sampled from a gamma distribution with a shape parameter of 0.4 in QMSim, and QTL jointly accounted for all additive genetic variation. During the 1095 generations of historical evolution, both QTL and SNP loci were simulated with a mutation rate of 2.5 × 10^−5^, and SNPs followed an “allele switching” mutation model without generating new alleles. The missing rate for marker genotypes was set to 0.01, and the genotyping error rate was set to 0.005. Parameters related to the simulated genome are summarized in Table 2.

#### 2.1.3. Genetic Evaluations

A single production trait representing beef cattle birth weight was simulated. Trait heritability was set to 0.42 and phenotypic variance to 1.0, consistent with reported estimates for birth weight in beef cattle [20]. The true breeding value (TBV) of each animal was calculated as the sum of additive effects across all QTL, as follows:

(1) $${TBV}_{k}=\sum_{j=1}^{qtl}\beta_{j}\cdot Q_{kj},$$

where qtl is the total number of QTLs, β_j_ is the additive effect of QTL j, and Q_kj_ is the QTL genotype of individual k at locus j (coded as 0, 1, or 2, representing the number of copies of a specific QTL allele carried by the individual). Phenotypes (yi) were simulated by adding residual terms, where the residuals were sampled as εi∼N(0, $\sigma_{e}^{2}$) and $\sigma_{e}^{2}$ is the residual variance.

EBV for all individuals in generations 9 and 10 of the current population were obtained from phenotypic records and pedigree information. Henderson’s mixed linear model [21] was used to compute BLUPs, which minimize the prediction error variance among all linear unbiased predictors. The following animal model was fitted:

(2) $$\left[Z^{\prime }Z+A^{-1}\frac{\sigma_{e}^{2}}{\sigma_{a}^{2}}\right]\left[\hat{a}\right]=\left[Z^{\prime }y\right]$$

where y is the vector of phenotypic values, Z is the incidence matrix relating phenotypic values to random additive effects (a), e is the vector of random errors, $\sigma_{a}^{2}\;$ is the additive genetic variance, and $\sigma_{e}^{2}\;$ is the residual variance. The mixed model was solved using the conjugate gradient method. For this birth weight–like trait, the TBVs and EBVs in each population followed approximately normal distributions with means close to 0, reflecting the simulation design with a fixed overall mean and homogeneous residual variance. The heritability was fixed at 0.42 in PopA, PopB and PopC. In all subsequent analyses, prediction accuracy was defined as the Pearson correlation between GEBVs and TBVs for this trait within each population and scenario.

### 2.2. Design of Genetic Distance Screening Strategies

Two genetic distance-based screening strategies were adopted in this study to construct cross-population reference sets, namely the Multidimensional Scaling (MDS) strategy and Fst-mediated stratified screening strategy. Both strategies took Population A as the core reference population and Populations B and C as candidate supplementary populations. Mixed reference populations were established by setting different genetic similarity thresholds (Top 10%, Top 15%, and Top 20%) to systematically evaluate the impact of varying genetic distance levels and mixing ratios on the prediction accuracy of GEBVs, and to determine the optimal individual introduction ratio and population structure.

#### 2.2.1. Design of MDS-Mediated Genetic Distance Screening

Individual genetic distances between PopA and PopB, as well as between PopA and PopC, were calculated using the MDS method. Individuals from PopB and PopC were ranked by genetic similarity in descending order, and the top 10%, 15%, and 20% of individuals with the highest genetic similarity to PopA were selected separately. These selected individuals were combined with all individuals from PopA to construct six mixed reference populations, with specific combinations as follows: A + 10%B, A + 15%B, A + 20%B, A + 10%C, A + 15%C, and A + 20%C. All genetic distance calculations and individual screening were performed using PLINKv1.9 software, and the accuracy of cross-population and within-population genomic prediction under different screening thresholds was evaluated based on the aforementioned reference populations.

#### 2.2.2. Design of Fst-Mediated Stratified Genetic Distance Screening

This strategy constructed cross-population reference sets using a four-step, stratified screening procedure. (1) Screening of highly differentiated SNPs: Pairwise Fst values were calculated between the target population (PopA) and each candidate reference population (PopB and PopC). SNPs with Fst > 0.1 were retained as highly differentiated loci, providing a core panel of markers for subsequent assessment of individual genetic similarity. (2) Calculation of Euclidean genetic distance: An individual genetic similarity matrix was built using the genotypes of the selected highly differentiated SNPs. For each individual in PopB and PopC, the genetic distance to individuals in PopA was defined as the Euclidean distance between their genotype vectors, thereby quantifying cross-population similarity at the individual level. (3) Selection of highly similar individuals: Individuals from PopB and PopC were ranked in ascending order of Euclidean distance (smaller distance indicating higher genetic similarity to PopA). The top 10%, 15%, and 20% of individuals in each population were selected to form subsets with different proportions of highly similar individuals. (4) Construction of cross-population reference sets: A stratified mixing scheme was applied. Each subset of highly similar individuals from PopB and PopC was combined with the original reference animals from PopA to generate six mixed reference populations, identical to those defined in the MDS strategy (A + 10%B, A + 15%B, A + 20%B, A + 10%C, A + 15%C, A + 20%C). Genomic prediction performance under these different combinations was then compared to identify the optimal strategy for cross-population genetic evaluation.

### 2.3. Functional Annotation and SNP Classification

To make the use of GO and KEGG annotations more transparent and reproducible, we designed a three-step integration workflow. Step 1 was resource selection. We used the Bos taurus ARS-UCD1.2 genome and bovine GO (Format-version: 1.2) and KEGG (Release 118, BR:br08601) databases, and we focused on terms related to growth, muscle development and reproduction, which are relevant for the simulated trait. Step 2 was the definition of functional regions. For each annotated gene, we defined a candidate region that included the gene body and 1 kb upstream and 1 kb downstream. Step 3 was SNP mapping and matrix construction. We mapped SNPs to these regions, generated GO-based and KEGG-based functional SNP sets, and built the corresponding G_func_ matrices for use in GBLUP, ssGBLUP and wGBLUP.

This study was based on the Bos taurus ARS-UCD1.2 reference genome, with GO (Gene Ontology Resource) and KEGG (KEGG: Kyoto Encyclopedia of Genes and Genomes) annotation datasets retrieved from their respective official databases, which served as the core annotation basis for screening functional SNPs. GO annotations included three major categories: biological process, cellular component and molecular function. KEGG annotations focused on metabolic pathways, signal transduction pathways, and various biological pathways associated with important economic traits of beef cattle, such as growth, muscle development and reproduction. This choice reflects a general principle of our framework, which is to prioritize annotation sources that match the species and the biology of the target trait. Raw annotation files were subjected to standardization to unify data formats and improve genomic annotation background information, laying a foundation for subsequent locus matching analyses.

Based on the Ensembl Bos taurus genome assembly database (ARS-UCD1.2), this study extracted chromosomal localization and genomic start and end site information of genes associated with each functional term (GO term or KEGG pathway). To account for the functional effects of potential regulatory regions of genes, the genomic boundaries of each gene were extended by 1 kb both upstream and downstream of the transcription start site, thus constructing SNP candidate regions covering gene coding regions and their upstream and downstream regulatory regions.

Chromosomal and physical position information of genome-wide SNPs was extracted from plink-formatted SNP map files. Genome-wide SNPs were matched with the aforementioned gene candidate regions through precise physical position alignment, and gene-associated SNPs were screened out. Based on the GO/KEGG pathway-gene correspondence, functional annotation information of genes was mapped to their associated SNPs to achieve hierarchical transfer of functional annotations. Gene-associated SNPs with clear GO/KEGG annotation information were defined as GO/KEGG-annotated functional SNPs. During the study, SNPs that could not be mapped to any gene or had no valid GO/KEGG annotations were excluded, and only SNPs with complete annotation information were retained. Two independent sets of functional SNPs were finally constructed: the GO-based functional SNP set contained 40,346 SNPs mapped to genes associated with GO biological processes, and the KEGG-based functional SNP set contained 16,586 SNPs mapped to genes associated with KEGG pathways. These datasets provided core data support for the subsequent construction of functional genomic relationship matrices.

Using the two screened GO and KEGG functional SNP sets as the basis, this study adopted the same genomic relationship calculation method as the standard GBLUP model to analyze pairwise genetic relationships among individuals in the study population. The calculated genetic relationship matrices were standardized, and then two specific functional genomic relationship matrices ($G_{func}$) based on GO-annotated and KEGG-annotated functional SNPs were constructed separately. These matrices provided a standardized genomic relationship basis for subsequent comparative analyses of multiple models.

### 2.4. Model and Analysis

The genomic prediction methods employed in this study included GBLUP, ssGBLUP, and wGBLUP. The key innovation across all models lies in the introduction of a $G_{func}$ matrix as a central parameter. The same integration framework can be applied to other annotation resources or species by replacing the functional SNP sets used to build $G_{func}$, while keeping the model structure unchanged. This matrix exclusively incorporates SNPs aligned with GO/KEGG functional annotations—specifically those associated with biological pathways or functions relevant to the target traits—thereby integrating biological prior knowledge to enhance both biological interpretability and predictive performance of the model.

#### 2.4.1. Genomic-Best Linear Unbiased Prediction

When leveraging functional genomic information for GBLUP parameter estimation, the analysis was conducted using a general linear mixed model developed with the HIBLUP software(v1.6.0). In this context, GBLUP utilizes a functional genomic relationship matrix ($G_{func}$) based on functionally annotated SNPs to GEBVs. The G_func_ matrix is defined as follows:

(3) $$G_{func}=\frac{{ZZ}^{\prime }}{\sum_{j=1}^{n}{2p}_{j}(1-p_{j})}$$

where Z represents the functional SNP genotype matrix (encoded as 0 for homozygotes, 1 for heterozygotes, and 2 for alternative homozygotes), n denotes the total number of SNP markers, j is the subscript of each functional SNP ranging from 1 to n, and $p_{j}$ is the allele frequency at the j-th SNP locus.

Since this study exclusively models additive genetic effects, only the variance structure for additive genetic effects is considered:

(4) $$Var\left(a\right)=G_{func}\sigma_{a}^{2}$$

The overarching linear mixed model equation for GBLUP is outlined below:

(5) y=Xb+Za+e

(6) $$\left[\begin{array}{cc} X^{\prime }X & X^{\prime }Z\\ Z^{\prime }X & Z^{\prime }Z+\lambda G_{func}^{-1} \end{array}\right]\left[\begin{array}{c} \hat{b}\\ \hat{a} \end{array}\right]=\left[\begin{array}{c} X^{\prime }y\\ Z^{\prime }y \end{array}\right]$$

In these equations, y denotes the vector of phenotypic observations, X is the design matrix that associates fixed effects with each animal, b represents the vector of fixed effects, Z is the functional SNP genotype matrix, a is the vector of additive genetic effects for individuals, $G_{func}$ is the functional genomic relationship matrix, e is the vector of residual error effects following a normal distribution ~N(0, $G\sigma_{e}^{2}$), $\sigma_{e}^{2}$ is the residual variance and $\lambda =\sigma_{e}^{2}/\sigma_{g}^{2}$.

#### 2.4.2. Single-Step Genomic Best Linear Unbiased Prediction

The statistical framework of the ssGBLUP model is consistent with GBLUP, with its core advantage lying in the construction of the blended relationship matrix H, which integrates pedigree information with functional genomic data to enable joint analysis of both genotyped and non-genotyped individuals. A key enhancement involves replacing the $G_{func}$ matrix in GBLUP with the blended relationship matrix H [22]. In our simulation, pedigree links between PopA, PopB and PopC were relatively weak. Therefore, the extra information that ssGBLUP could borrow from non-genotyped relatives across populations was limited. In practical breeding programs, ssGBLUP is expected to show a clearer advantage when many non-genotyped animals with phenotypes are closely related to genotyped candidates through well-connected pedigrees. The inverse of the H matrix is expressed as follows:

(7) $$H^{-1}=A^{-1}+\left[\begin{array}{cc} 0 & 0\\ 0 & G_{func}^{-1}-A_{22}^{-1} \end{array}\right]$$

where $H^{-1}$ represents the inverse of the matrix that amalgamates both pedigree and genomic data, $G_{func}^{-1}$ denotes the inverse of the functional genomic relationship matrix, and $A_{22}^{-1}$ corresponds to the inverse of the numerator relationship matrix specific to genotyped animals. The $G_{func}$ matrix is constructed as per the formula outlined in Equation (3).

#### 2.4.3. Weighted Best Linear Unbiased Prediction

The core improvement in the wGBLUP lies in assigning differential weights to functional SNPs, thereby enhancing the contribution of high-effect SNPs to genetic relationship estimation. While maintaining the same model framework as GBLUP, wGBLUP introduces SNP-specific weights during the construction of the genomic relationship matrix. Using the SLEMM software (v0.90.1) [23], this study implemented a weighting scheme based on SNP effect estimates to optimize genomic prediction.

SLEMM fitted the following linear mixed model:

y=Xβ+Zα+e

(8) $$\alpha \sim N(0,W\sigma_{\alpha }^{2})$$

$$e\sim N(0,R\sigma_{e}^{2})$$

where y represents the vector of phenotypes for a quantitative trait, β encapsulates fixed effects including the mean, X serves as the design matrix for β, α denotes the vector of SNP effects with a diagonal covariance matrix $W\sigma_{\alpha }^{2}$, Z is the matrix of standardized genotypes, and e is the vector of residuals with a diagonal covariance matrix $R\sigma_{e}^{2}$. The diagonal elements of W are weights, where each weight signifies the relative impact of the corresponding SNP on genetic variance, i.e., Wj indicates the SNP j’s contribution to genetic variance.

Given the tendency for nearby SNP loci to capture the effects of a QTL due to LD, and the similarity in the effects of adjacent SNPs in model fitting, this study employs a second SNP weighting scheme, defined as:

(9) $$W_{jj}=C\cdot \frac{1}{2S+1}\sum_{k-j-S}^{j+S}\hat{\alpha_{k}^{2}}$$

where C is a scaling constant to control the mean weight to be 1, S is the number of SNPs on each side of SNP j, and $\hat{\alpha_{k}}$ is the estimated effect of the kth SNP from a pre-existing BLUP model with W equivalent to the identity matrix. This SNP’s weight derivation borrows information from a surrounding window of 2S + 1 SNPs. SLEMM initially fits the model with training data where W is the identity matrix, followed by fitting with W computed as per Equation (9).

Based on the aforementioned weights, a weighted functional genomic relationship matrix $G_{w,func}$ was constructed as follows:

(10) $$G_{w,func}=\frac{{ZWZ}^{\prime }}{\sum_{j=1}^{n}{w_{j}2p}_{j}(1-p_{j})}$$

In the wGBLUP model, we adopted the same strategy as in GBLUP, replacing only the $G_{func}$ matrix with $G_{w,func}$. The weight updates strictly followed the default SLEMM procedure (Equation (9)), with no additional priors imposed on functional SNPs, to evaluate whether matrix substitution alone is sufficient to capture pathway information.

### 2.5. Evaluation of Genomic Prediction Accuracy and Unbiasedness

In this study, model performance was evaluated in terms of predictive accuracy and fairness (unbiasedness). Because TBV were known in the simulation, predictive accuracy was quantified as the Pearson correlation between GEBV and TBV:

Accuracy = Corr(GEBV, TBV)(11)

where the correlation coefficient ranges from 0 to 1 and reflects the strength of the linear association between GEBV and TBV.

Fairness of the predictions was assessed from the regression of GEBV on TBV:

b = Cov(GEBV, TBV)/Var(TBV)(12)

where b is the regression coefficient. A regression coefficient close to 1 and an intercept close to 0 indicates that the genomic predictions are essentially unbiased.

### 2.6. Two-Way Analysis of Variance (Two-Way ANOVA)

To evaluate differences in genomic prediction accuracy under different population structures and experimental settings, we applied a two-way analysis of variance (two-way ANOVA). Statistical analyses were conducted in Prism (v8.0.2). Two main factors were considered: (1) population type (e.g., single-breed vs. multi-breed populations) and (2) experimental condition (e.g., different mixing proportions or evaluation models). For each factor and their interaction, F-statistics and corresponding *p*-values were calculated. A *p*-value < 0.05 was used as the criterion for statistical significance.

## 3. Results

### 3.1. Comparative Assessment of Cross-Breed Genomic Prediction with GO-Integrated GBLUP Models

**Figure 2:**
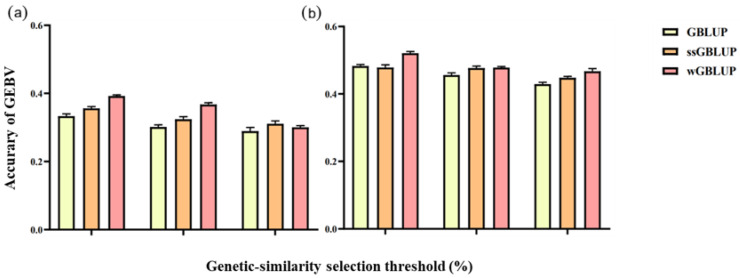
Comparison of genomic prediction accuracy of different models constructed by GO-annotated SNP sets based on IBS mixed reference populations: (**a**) PopB; (**b**) PopC. The values are expressed as the mean ± SE (standard error).

In composite reference populations at different genetic relatedness levels, all three genomic prediction models (GBLUP, ssGBLUP, wGBLUP) showed significantly higher overall prediction accuracy for PopB than for PopA. The wGBLUP model integrated with GO annotation information maintained the optimal predictive performance in both PopA and PopB (Figure 2). For PopA, the target population (PopB or PopC) accounted for 10% of the composite reference population. The prediction accuracy of the wGBLUP model was approximately 0.40, significantly higher than GBLUP (≈0.35) and ssGBLUP (≈0.38). When the proportion increased to 20%, the prediction accuracy of the wGBLUP model decreased to about 0.35, but it was still higher than the GBLUP and ssGBLUP models at the same proportion. PopB had closer genetic relatedness, and its overall prediction accuracy improved more significantly. At a 10% proportion, the prediction accuracy of the wGBLUP model reached approximately 0.55, higher than GBLUP (≈0.50) and ssGBLUP (≈0.52). When the proportion rose to 20%, the prediction accuracies of the three models all remained between 0.45 and 0.50. The wGBLUP model still retained a relative advantage.

**Figure 3:**
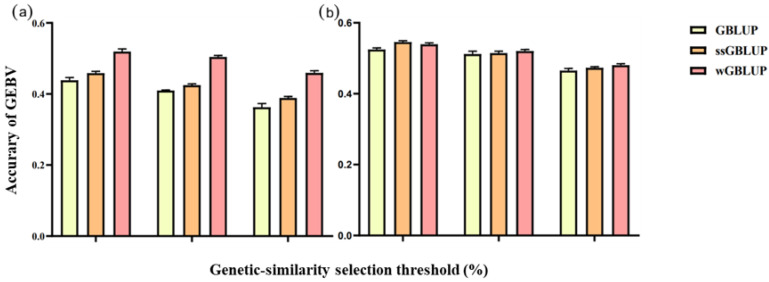
Comparison of genomic prediction accuracy of different models constructed by GO-annotated SNP sets based on FST mixed reference populations: (**a**) PopB; (**b**) PopC. The values are expressed as the mean ± SE (standard error).

In composite reference populations with different genetic differentiation distances, the predictive advantage of the wGBLUP model persisted. PopB with low genetic differentiation had significantly higher overall prediction accuracy than PopA with high genetic differentiation (Figure 3). For PopA, the target population accounted for 10% of the composite reference population. The prediction accuracy of the wGBLUP model was approximately 0.50, higher than GBLUP (≈0.45) and ssGBLUP (≈0.48). When the proportion increased to 20%, the prediction accuracy of the wGBLUP model decreased to about 0.45, but it remained the leading model. For PopB, the prediction accuracy of the wGBLUP model peaked at approximately 0.60 at a 10% proportion. This value was significantly higher than GBLUP (≈0.55) and ssGBLUP (≈0.58). When the proportion rose to 20%, the prediction accuracies of the three models tended to converge. They all remained between 0.45 and 0.50, and the wGBLUP model was still optimal. Notably, as the composite reference population size expanded, genetic relatedness between populations became closer, and the genetic differentiation distance was reduced. The wGBLUP model integrated with GO annotation information showed significantly greater improvements in prediction accuracy and unbiasedness than the GBLUP and ssGBLUP models. This confirmed the positive regulatory effect of GO annotation information on cross-breed genomic prediction. It also verified the adaptive advantage of the weighted model in complex population structures.

Notably, the wGBLUP model integrated with GO annotation information was tested in specific scenarios. As the population size expanded, genetic relatedness became closer, and the genetic differentiation distance was reduced. Under such scenarios, this model achieved greater gains in prediction accuracy and unbiasedness relative to GBLUP and ssGBLUP. The result demonstrates that GO annotation information can effectively boost cross-breed genomic prediction performance, and verifies that weighted models possess better applicability for genetically complex populations.

### 3.2. Comparative Assessment of Cross-Breed Genomic Prediction with KEGG-Integrated GBLUP Models

**Figure 4:**
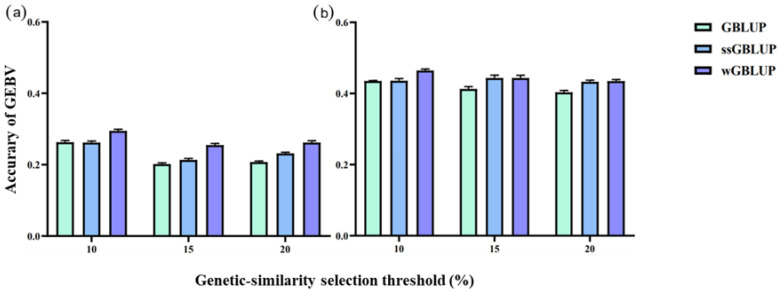
Comparison of genomic prediction accuracy of different models constructed by KEGG-annotated SNP sets based on IBS mixed reference populations: (**a**) PopB; (**b**) PopC. The values are expressed as the mean ± SE (standard error).

Evaluation models were constructed with KEGG-annotated SNP sets. In these models, the wGBLUP model maintained high prediction accuracy across composite reference populations at different genetic relatedness levels. The overall predictive performance of PopB was significantly better than that of PopA (Figure 4). For PopA, the target population accounted for 10% of the composite reference population. The prediction accuracy of the wGBLUP model was approximately 0.35. It was higher than for GBLUP (≈0.28) and ssGBLUP (≈0.30). When PopA accounted for 10% of the composite reference population, the prediction accuracy of the wGBLUP model was approximately 0.35. Even so, it was still the highest among the three models. When PopB accounted for 10% of the reference population, the prediction accuracy of the wGBLUP model reached approximately 0.45, which was higher than for GBLUP (≈0.42) and ssGBLUP (≈0.43). When the proportion rose to 20%, the prediction accuracies of the three models all remained between 0.40 and 0.45. The advantage of the wGBLUP model was still obvious.

**Figure 5:**
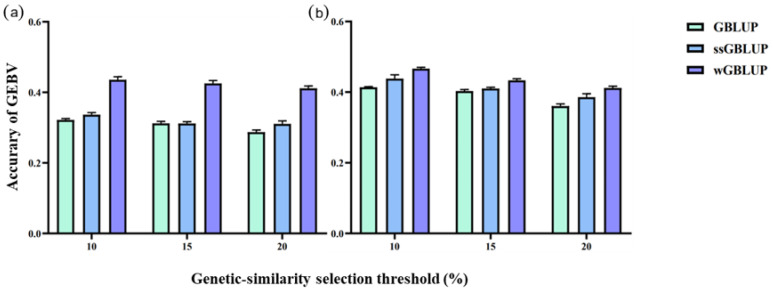
Comparison of genomic prediction accuracy of different models constructed by KEGG-annotated SNP sets based on FST mixed reference populations: (**a**) PopB; (**b**) PopC. The values are expressed as the mean ± SE (standard error).

In composite reference populations with different genetic differentiation distances, the wGBLUP model showed the highest prediction accuracy in both PopA and PopB. PopB with low genetic differentiation had significantly better overall predictive performance than PopA with high genetic differentiation (Figure 5). For PopA, the target population accounted for 10% of the composite reference population. The prediction accuracy of the wGBLUP model was approximately 0.45. It was higher than for GBLUP (≈0.35) and ssGBLUP (≈0.38). When the proportion increased to 20%, the prediction accuracy of the wGBLUP model decreased to about 0.42. For PopB, the target population proportion was 10%. The prediction accuracy of the wGBLUP model reached approximately 0.48. It was higher than for GBLUP (≈0.42) and ssGBLUP (≈0.43). When the proportion rose to 20%, the prediction accuracies of the three models clustered between 0.40 and 0.45. The wGBLUP model was still optimal. Notably, the wGBLUP model integrated with KEGG pathway information showed obvious advantages. This was especially true when the composite reference population size expanded, genetic relatedness became closer, and genetic differentiation distance was reduced. In these scenarios, the wGBLUP advantage in prediction accuracy was more prominent. Its prediction bias was also closer to the ideal value (0) overall. This result highlights the positive role of KEGG annotation information in cross-breed genomic prediction. It also verifies the stability advantage of the weighted model in complex population scenarios.

Incorporating KEGG pathway information noticeably improved the practical performance of wGBLUP. As the reference population expanded and genetic divergence decreased, the model maintained stable predictive accuracy and desirable unbiasedness. The finding proves pathway annotation can facilitate inter-breed genomic evaluation, and weighted modeling fits well for heterogeneous population analysis.

### 3.3. Comparative Performance of KEGG vs. GO Functional Annotations in Multi-Breed Prediction

**Figure 6:**
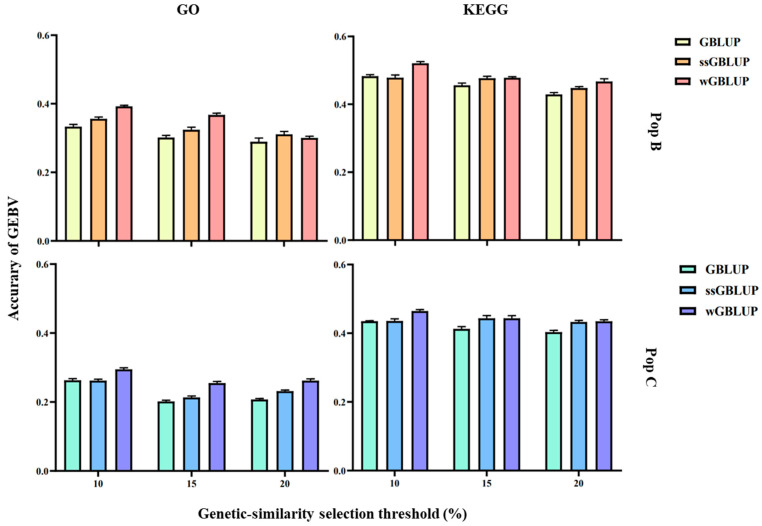
Prediction accuracy of different evaluation models for simulated two-population datasets in composite reference populations at different genetic relatedness levels. Note: GO represents different evaluation models constructed with GO-annotated SNP sets; KEGG represents different evaluation models constructed with KEGG-annotated SNP sets. The values are expressed as the mean ± SE (standard error).

In composite reference populations at different genetic relatedness levels, the wGBLUP models built with GO and KEGG annotation data all followed the same rule. The closer the genetic relatedness between populations, the higher the prediction accuracy. However, the applicable scenarios of these two functional annotation data differed significantly (Figure 6). In PopB with closer genetic relatedness, the GO-wGBLUP model had a notable advantage in accuracy. At a 10% target population proportion, its prediction accuracy reached 0.55, significantly higher than the 0.45 of KEGG-wGBLUP under the same conditions. In PopA with relatively distant genetic relatedness, the KEGG-wGBLUP model showed stronger stability and a smaller decline in prediction accuracy. When the population proportion increased from 10% to 20%, the accuracy of KEGG-wGBLUP dropped from 0.35 to 0.28, a 20% reduction. This decline was larger than the 12.5% reduction in GO-wGBLUP, whose accuracy decreased from 0.40 to 0.35. In addition, under the GO annotation system, the accuracy difference between PopB and PopA was 0.15 at a 10% proportion. Under the KEGG annotation system, this difference was 0.10. This suggested that GO annotation data were more sensitive to the genetic relatedness between populations.

**Figure 7:**
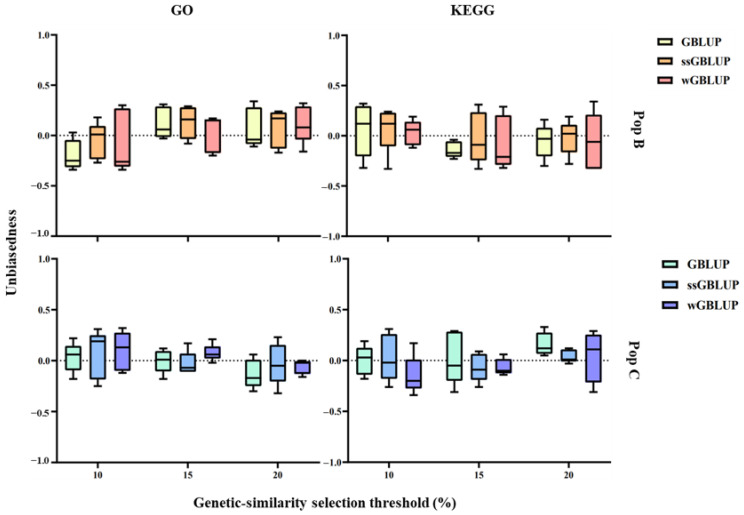
Prediction bias of different evaluation models for simulated two-population datasets in composite reference populations at different genetic relatedness levels. Note: GO represents different evaluation models constructed with GO-annotated SNP sets; KEGG represents different evaluation models constructed with KEGG-annotated SNP sets. The values are expressed as the mean ± SE (standard error).

Results in Figure 7 showed that in composite reference populations at different genetic relatedness levels, the wGBLUP models constructed with GO and KEGG-annotated SNP sets both had better prediction unbiasedness than the GBLUP and ssGBLUP models (their bias values were closer to the ideal value of 0). Among them, the KEGG-wGBLUP model exhibited stronger stability and a smaller bias fluctuation range in PopC with relatively distant genetic relatedness. In PopB with closer genetic relatedness, there was no significant difference in bias between the wGBLUP models corresponding to the two types of annotation information. However, both models were significantly superior to the traditional GBLUP and ssGBLUP models. This confirmed the positive effect of functional annotation information on reducing the bias of cross-population prediction.

**Figure 8:**
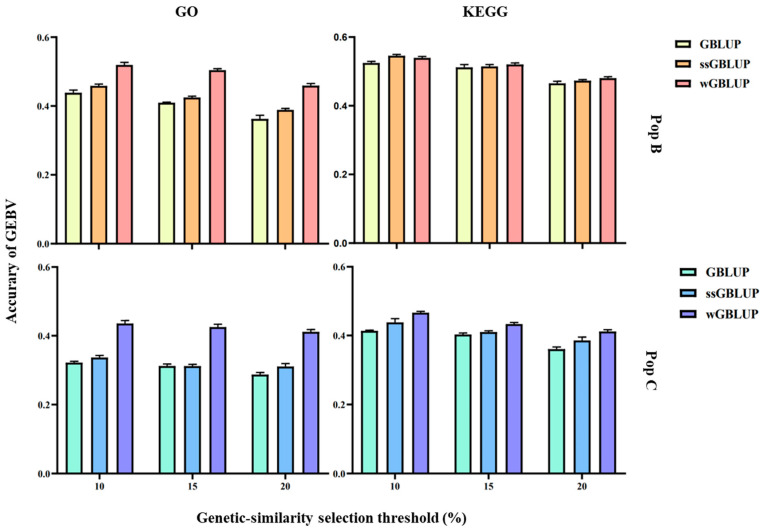
Prediction accuracy of different evaluation models for simulated two-population datasets in composite reference populations at different genetic differentiation distance levels. Note: GO represents different evaluation models constructed with GO-annotated SNP sets; KEGG represents different evaluation models constructed with KEGG-annotated SNP sets. The values are expressed as the mean ± SE (standard error).

In composite reference populations with different genetic differentiation distances, both GO and KEGG functional annotation information effectively mitigated the negative impact of genetic differentiation on predictive performance. KEGG pathway information, however, showed stronger adaptability in populations with high genetic differentiation (Figure 8). In PopB with low genetic differentiation, the GO-wGBLUP model still maintained a higher peak accuracy. At a 10% target population proportion, its prediction accuracy reached 0.60, which was higher than the 0.48 of KEGG-wGBLUP. In PopA with high genetic differentiation, the accuracy advantage of the KEGG-wGBLUP model gradually became prominent. At a 10% proportion, the prediction accuracy of KEGG-wGBLUP was 0.45, with a difference of only 0.05 from the 0.50 of GO-wGBLUP. When the proportion increased to 20%, the reduction in prediction accuracy of KEGG-wGBLUP was only 6.7%, significantly lower than the 10% reduction in GO-wGBLUP. Meanwhile, the difference in prediction accuracy between PopB and PopA under the GO annotation system was 0.10, while the difference under the KEGG annotation system was only 0.03. This indicated that KEGG pathway information had higher tolerance to the genetic differentiation distance between populations, and was more suitable for cross-breed genomic prediction in the context of high genetic differentiation.

**Figure 9:**
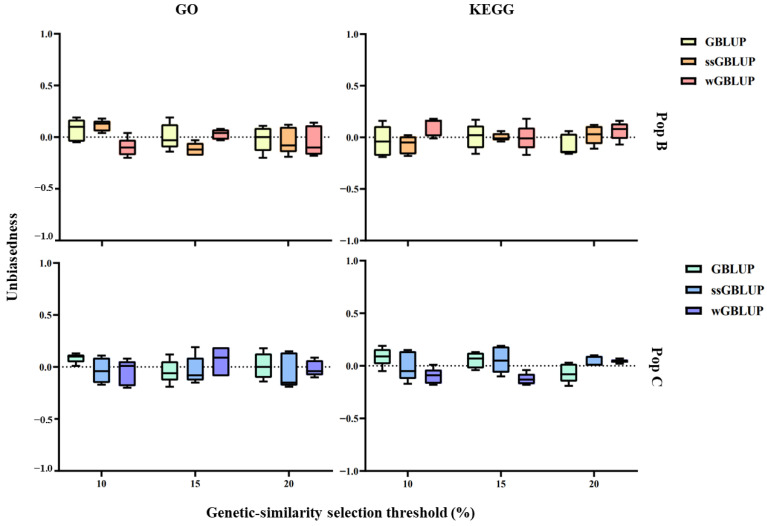
Prediction of different evaluation models for simulated two-population datasets in composite reference populations at different genetic differentiation distance levels. Note: GO represents different evaluation models constructed with GO-annotated SNP sets; KEGG represents different evaluation models constructed with KEGG-annotated SNP sets. The values are expressed as the mean ± SE (standard error).

Figure 9 shows the prediction bias of GBLUP, ssGBLUP and wGBLUP models constructed with GO and KEGG-annotated SNP sets in composite reference populations at different genetic differentiation distance levels. The ideal value of bias is 0, and values closer to 0 indicate better unbiasedness. Overall, at the three population proportions of 10%, 15% and 20%, the wGBLUP models corresponding to the two functional annotations all yielded better unbiasedness than the traditional GBLUP and ssGBLUP models. Their bias values were closer to 0 with a smaller fluctuation range. Among them, the KEGG-wGBLUP model showed more tolerance to genetic differentiation distance. In populations with high genetic differentiation, such as PopA, its bias remained stable with no obvious increase, even when the population proportion increased from 10% to 20%. In contrast, the GO-wGBLUP model had a slight fluctuation in bias with the increase in population proportion under the scenario of high genetic differentiation, but it was still superior to the GBLUP and ssGBLUP models under the same conditions. In populations with low genetic differentiation, such as PopB, there was a small difference in bias between the GO-wGBLUP and KEGG-wGBLUP models, and both were significantly better than the traditional models. This further confirmed that functional annotation information could effectively reduce the bias of cross-population prediction. Moreover, the weighted model (wGBLUP) had stronger adaptability to different genetic differentiation scenarios after integrating functional annotation information.

## 4. Discussion

GS, the core technology of modern breeding, establishes prediction equations using reference populations with complete phenotypic and genotypic information to achieve accurate selection of candidate individuals. Compared with traditional breeding methods, the remarkable advantages of GS in improving prediction accuracy, accelerating genetic progress and optimizing breeding efficiency have been well verified by numerous theoretical and experimental studies [24,25,26].

However, the application of GS in the breeding of minor beef cattle breeds is significantly limited, with the core bottleneck being the difficulty in establishing large reference populations with accurate phenotypic data. In this regard, multi-breed data integration or cross-breed prediction is regarded as an effective remedial strategy, which involves introducing data from other breeds to optimize SNP effect estimation or conducting cross-population prediction using SNP effects from large populations [5]. Although the integration of multi-population reference populations is expected to improve prediction accuracy, this strategy still faces challenges such as insufficient capture of genetic variation and interference from population dominance: single-population references tend to reduce prediction accuracy due to failure to cover the genetic variation in target populations, and blind expansion of sample size may introduce genetic noise and weaken the generalization ability of models [27,28].

The integration of functional annotation information provides a new approach to address the above challenges, as it can effectively narrow the search range for causal variants and enhance the robustness of cross-population prediction models [29]. Based on recent advances in functional genomics research, GO and KEGG annotations enable accurate screening of functional SNPs from the perspectives of functional classification and pathway regulation. This reduces interference from neutral markers and improves the biological interpretability of models simultaneously [30,31]. Existing studies have confirmed that the functional annotation integration strategy shows promising application prospects in livestock and poultry breeding, providing an important reference for multi-population GS research in beef cattle. Innovations in the architecture of genomic prediction models are mostly based on the optimization of traditional GBLUP models, with the core being the differential treatment of functional markers and conventional markers to achieve in-depth integration of functional biological information and statistical modeling. In sheep breeding, the BayesRC model classifies functional markers separately for modeling, which significantly improves predictive performance: compared with the traditional 50K chip model, the prediction accuracy is increased by 8.7–10.2%. Further validation in Australian multi-breed sheep populations shows that for nine key economic traits including post-weaning weight and eye muscle depth, this model increases the average prediction accuracy by 0.102 and 0.087 in purebred Merino and crossbred Border Leicester × Merino populations with low genetic correlation, respectively. Improvement is greater for traits with complex genetic architectures, such as shear force and intramuscular fat [32].

In dairy cattle research, the dimensionality of functional marker classification in the BayesRC model has been further expanded: studies divided variants based on six categories of functional annotations including ChIP-seq peak regions and cross-species conserved sites, and constructed prediction models combined with high-density chips. In breeds such as Holstein and Jersey, for functional traits including mastitis and somatic cell score, the prediction accuracy of models integrating conserved sites was 2.1–3.5% higher than that of the traditional high-density GBLUP model; this advantage was particularly prominent in Australian Red cattle with a small population size (only 91 validation individuals), confirming that functional annotation can effectively make up for the limitations of prediction in small populations. Other studies addressed the issue of genetic heterogeneity in multi-breed GS for dairy cattle by screening cross-breed shared predictive variants and integrating functional annotation information, which increased the cross-breed prediction accuracy of traits such as milk yield and milk protein percentage in Holstein, Jersey and other breeds by 3.2–5.7%, with a more significant improvement in Jersey cattle with a small reference population (only 1200 individuals) [33]. In addition, screening evolutionarily constrained functional variants using whole-genome sequence data and combining multi-breed joint modeling increased the cross-breed prediction accuracy of dairy cattle reproductive traits by 8.3–10.1% compared with traditional SNP chip data, highlighting the advantages of functional sequence variants in overcoming genetic barriers between breeds [34].

In swine breeding research, the functional annotation integration strategy has also demonstrated favorable application value. Jiang et al. [35] adopted a strategy combining GWAS preselected variants with the weighted BLUP model, and improved the prediction accuracy of traits such as average daily gain and backfat thickness by 0.3–2.0% through the construction of a weighted genomic relationship matrix. This model screened 1–5% of core variants based on multi-population GWAS and dynamically adjusted weights according to effect values. Among three commercial lines, the weight optimization effect was significant for high-heritability traits such as backfat thickness; in particular, in the LEPR region on chromosome 12, the proportion of genetic variance explained by the weighted model reached 8.7%, which was much higher than the 4.3% of the traditional model. Other studies optimized genomic prediction models by integrating multi-population data with functional annotation prior knowledge (such as metabolic pathway and QTL region information), which increased the prediction accuracy of traits including average daily gain and lean meat percentage in Duroc, Landrace and Yorkshire three-way cross populations by 2.1–3.8%. Meanwhile, the prediction robustness for crossbred progeny of different lines was significantly enhanced, effectively alleviating the prediction bias caused by genetic background differences in multi-line joint breeding [36]. Aiming at the problems of small population size and limited genetic resources in some minor local breeds, the use of cross-breed shared SNP effects combined with functional annotation to screen core variants increased the genomic prediction accuracy of growth traits in minor local breeds by 5.1–7.3%, providing a feasible technical approach for the genetic improvement in minor breeds [37].

All the aforementioned studies have confirmed that the core value of functional annotation lies in the accurate identification of cross-breed conserved causal variants and the reduction in noise interference arising from genetic background heterogeneity and neutral variants. Compared with traditional equal-weight models, independently optimizing functional markers—using methods such as class-weighted Bayesian modeling or multi-population joint modeling—produces substantially stronger results. These advantages are especially evident in cross-breed prediction, small population breeding, and the enhancement of lowly heritable traits. This provides an effective solution to key breeding challenges, including distant population prediction and genetic improvement in minor breeds. Based on the GBLUP, ssGBLUP and wGBLUP models, this study reconstructed the G matrix using GO/KEGG-annotated SNPs, and systematically evaluated their cross-population prediction performance in beef cattle populations with varying levels of genetic relatedness and differentiation. The conclusions drawn are largely consistent with those of the above studies, and further refine the adaptation rules of functional annotation for cross-population scenarios in beef cattle breeding.

Our results showed that SNP sets annotated with GO and KEGG both improved the performance of cross-population prediction in beef cattle, and the wGBLUP model exhibited the most stable performance after integrating functional annotations. Meanwhile, the combination of functional annotations and the weighted model markedly reduced the bias of GEBVs, with the corresponding deviation values closer to the ideal value of 0. Specifically, in PopB with low genetic differentiation and close genetic relatedness, the GO-wGBLUP model performed better: at a 10% population proportion, the prediction accuracy reached 0.55~0.60, which was significantly higher than that of GBLUP (approximately 0.50) and ssGBLUP (approximately 0.52), and the peak accuracy reached 0.60, significantly higher than the 0.45 of KEGG-wGBLUP under the same conditions. In contrast, in PopA with high genetic differentiation and distant genetic relatedness, the KEGG-wGBLUP model demonstrated stronger stability: when the population proportion increased from 10% to 20%, its prediction accuracy decreased from 0.45 to 0.42 with a reduction of only 6.7%, much lower than the 10% reduction in GO-wGBLUP (from 0.50 to 0.45) and 20% reduction in the traditional GBLUP model. Additionally, the accuracy difference between PopA and PopB under the KEGG annotation system was only 0.03, far lower than the 0.10 under the GO annotation system. This pattern was also observed in PopC, where the KEGG-wGBLUP model outperformed the GO-wGBLUP model at all population proportions, highlighting the anti-interference ability of pathway modules.

In terms of bias performance, the wGBLUP models corresponding to the two functional annotations were both significantly superior to GBLUP and ssGBLUP. The KEGG-wGBLUP model exhibited extremely strong stability in PopC with distant genetic relatedness and PopA with high genetic differentiation, with a bias fluctuation range of less than 0.05. In PopB with close genetic relatedness, the GO-wGBLUP model showed no significant difference from the KEGG-wGBLUP model, but the fluctuation range of its bias increased slightly under high genetic differentiation scenarios. This result indicates that functional annotation information can not only improve prediction accuracy but also reduce the bias of GEBVs, and there are distinct differences in the applicable scenarios of different annotation systems, providing a basis for the precise selection of models in practical breeding.

In addition, this study observed a decrease in predictive performance with the expansion of the composite reference population size, which was fully consistent with the preset hypothesis—that blind increases in sample size in cross-population scenarios introduce redundant population-specific genetic information. Notably, however, the performance reduction in models integrated with functional annotations was significantly smaller than that of traditional models: taking PopA as an example, the accuracy of GO-wGBLUP decreased from 0.40 to 0.35 with a reduction of only 12.5%, whereas that of GBLUP dropped from 0.35 to 0.28 with a reduction of up to 20%. This indicates that SNP sets with functional annotations can effectively filter out noise information irrelevant to target traits and enhance the generalization ability of models, which is consistent with the research conclusions of Zhao et al. [38] in cross-population prediction of pigs. Meanwhile, the ssGBLUP model consistently exhibited performance between GBLUP and wGBLUP in this study and did not show significant advantages. It is speculated that this is due to the weak pedigree correlation between populations in cross-population scenarios, making it difficult for ssGBLUP to exert its advantage of relying on pedigree-integrated information. In contrast, wGBLUP strengthens core genetic signals by weighting functional SNPs, making it more suitable for complex population structures.

This difference in predictive performance stems from the distinct core characteristics of the GO and KEGG annotation systems, which are mainly reflected in information dimension, aggregation level, trait association mode and evolutionary conservation. GO annotation primarily classifies the functions of individual genes [39], making it suitable for the precise identification of genes directly associated with traits. In populations with similar genetic backgrounds, such information can effectively capture key genetic signals. However, GO contains a large number of functional terms, and related genes may be scattered across different terms. In populations with high genetic differentiation, linkage disequilibrium is prone to disruption, causing some markers to lose their predictive effect and thus leading to a more pronounced decline in the accuracy of GO-wGBLUP. In contrast, KEGG annotation integrates the interactions of multiple genes at the pathway level to systematically reveal the regulatory networks underlying traits [40]. Such pathway-based information is insensitive to the variation in individual genes and clusters functionally related genes into the same module, forming regions with concentrated genetic effects. Even if the linkage relationships of some markers change across different populations, other markers within the pathway can still provide supplementary information, thereby maintaining predictive stability. To our knowledge, no previous study has reported the specific pattern observed here: GO-based models perform better in closely related groups, whereas KEGG-based models perform better in more distantly related groups. However, several studies in dairy cattle and pigs have suggested that pathway-level annotations tend to show more robust cross-population performance than gene-level annotations, which is broadly consistent with our findings. Therefore, we interpret the pattern observed here as a plausible but still preliminary result. It may also be influenced by species, trait types, and the completeness of GO and KEGG databases, and thus needs to be validated in additional populations and datasets.

Despite the clear conclusions drawn from this study, certain limitations remain. First, functional annotation relies on the completeness of existing databases, and some beef cattle-specific functional genes or pathways may not be fully annotated, which affects the coverage of functional SNP sets. This incomplete coverage may cause some truly functional variants to be treated as neutral SNPs in our models, which can attenuate the advantage of GO- or KEGG-based SNP sets and partly bias the comparison between them. As a result, some pathway or gene effects may be underestimated rather than incorrectly detected. In future work, this limitation could be mitigated by incorporating additional annotation resources, by transferring annotations from well-studied species through orthology, and by integrating transcriptomic or epigenomic data from relevant tissues. Second, this study focused on three population size proportions and specific levels of genetic differentiation, and the generalizability of the conclusions needs to be further verified by expanding the parameter range. Therefore, our parameter space covers only a subset of realistic breeding scenarios. The robustness and transferability of our conclusions may change under more extreme conditions, for example, when the reference population is much smaller or much larger, or when the genetic differentiation between populations is very low or very high. Under these situations, the relative benefits of GO or KEGG annotations and of the wGBLUP model may increase or decrease. Future simulation and empirical studies with a broader grid of population sizes and genetic differentiation levels will be needed to evaluate how general our conclusions are. Based on these points, future research can further screen core functional genes by integrating transcriptome data or optimize the model architecture with epigenetic information, aiming to further improve the performance of cross-population genomic prediction in beef cattle and provide more comprehensive technical support for multi-population joint breeding.

## 5. Conclusions

This study evaluated GBLUP, ssGBLUP and wGBLUP models that integrate GO and KEGG annotations for cross-population genomic prediction in beef cattle. Functionally annotated SNPs increased the accuracy and stability of cross-population prediction and reduced genetic noise in mixed reference populations. Among the three models, the annotated wGBLUP model showed the most robust performance and the smallest bias of GEBVs. In the simulated scenarios, GO-based models performed better in populations with close genetic relationships, whereas KEGG-based models performed better in populations with high genetic differentiation. Weak pedigree connections between populations limited the additional benefits of ssGBLUP. These results indicate that wGBLUP combined with suitable functional annotations is a promising choice for multi-population genomic selection. GO- or KEGG-based annotations can be selected according to population structure and genetic distance. The patterns reported here are based on a single birth-weight-like trait and three hypothetical beef cattle populations, so they should be considered trait- and scenario-specific. Validation across more traits and cattle breeds, including both local and commercial populations, is required to evaluate the general applicability of these findings. This study also outlines a simple framework that could be extended to other types of biological information. In future work, transcriptomic or epigenetic data from relevant tissues may be used to define alternative functional SNP sets and corresponding genomic relationship matrices within the same modeling scheme. When this framework is applied to real breeding populations, practical issues such as imperfect phenotypes, genotyping errors and incomplete annotations may reduce the observed gains. Evaluation of real data will be necessary to assess how robust the conclusions are under such conditions.

## Figures and Tables

**Table 1:** Parameters of the simulation process.

Parameter	PopA	PopB	PopC
Step 1: Historical Generations (HG)			
Number of generations phase 1 (size)	0 (200)	0 (500)	0 (1000)
Number of generations phase 2 (size)	1000 (200)	1000 (200)	1000 (1000)
Number of generations phase 3 (size)	1095 (1000)	1095 (1000)	1095 (200)
Step 2: Expansion Generations (EG)			
Number of founder males from HG	250		
Number of founder females from HG	3000	2550	3000
Number of generations	10		
Number of offspring per dam	1		
Mating system	Random		
Step 3: Recent Generations (RG)			
Number of founder males from EG	50	55	50
Number of founder females from EG	2000	2100	2200
Number of generations	100		
Number of offspring per dam	1		
Selection/culling basis	Phenotype/BLUP-EBV		
Sire replacement rate	60%	50%	50%
Dam replacement rate	30%	30%	20%
Rate of missing sire/dam records	0.05		

**Table 2:** Parameters of the simulated genome.

Parameter	PopA	PopB	PopC
Number of chromosomes	29 (no X Chr)		
Genome length	2715.85 cM		
Number of markers	50 k		
Marker/QTL positions	Random		
Number of marker/QTL alleles	2/2 3 4		
Marker allele frequencies	Equal		
QTL allele effects	Equal		
Additive allelic effects for QTL	Gamma distribution (shape = 0.4)		
Rate of missing marker genotypes	0.01		
Rate of marker genotyping error	0.005		
Rate of recurrent mutation	0.0001		

## Data Availability

The data analyzed in this study were generated through simulation and are not deposited in a public repository. Detailed descriptions of the simulation parameters and procedures are provided in the “Materials and Methods” section of this manuscript. The simulation was conducted using publicly accessible software, which can be found at https://animalbiosciences.uoguelph.ca/~msargol/qmsim/ (accessed on 8 October 2025). Upon reasonable request, the corresponding author will provide any additional data necessary for the replication of the study’s findings.

## Abbreviations

The following abbreviations are used in this manuscript:

GS	Genomic selection
TBV	True breeding value
EBV	Estimated Breeding Value
BLUP	Best linear unbiased prediction
GEBV	Genomic estimated breeding value
SNP	Single-nucleotide polymorphism
MAF	Minor allele frequency
LD	Linkage disequilibrium
QTL	Quantitative trait loci
GBLUP	Genomic best linear unbiased prediction
ssGBLUP	Single-step genomic best linear unbiased prediction
wGBLUP	Weighted genomic best linear unbiased prediction
GRM	Genomic relationship matrix
GO	Gene Ontology
KEGG	Kyoto Encyclopedia of Genes and Genomes
